# Nanoelectromechanical resonant narrow-band amplifiers

**DOI:** 10.1038/micronano.2016.4

**Published:** 2016-03-24

**Authors:** Alireza Ramezany, Mohammad Mahdavi, Siavash Pourkamali

**Affiliations:** 1 Department of Electrical Engineering, University of Texas at Dallas, Richardson, TX 75080, USA

**Keywords:** NEMS, nanoelectromechanical amplifier, narrow-band filtering, displacement amplification, thermal piezoresistive resonance, effective quality factor improvement

## Abstract

This study demonstrates amplification of electrical signals using a very simple nanomechanical device. It is shown that vibration amplitude amplification using a combination of mechanical resonance and thermal-piezoresistive energy pumping, which was previously demonstrated to drive self-sustained mechanical oscillation, can turn the relatively weak piezoresistivity of silicon into a viable electronic amplification mechanism with power gains of >20 dB. Various functionalities ranging from frequency selection and timing to sensing and actuation have been successfully demonstrated for microscale and nanoscale electromechanical systems. Although such capabilities complement solid-state electronics, enabling state-of-the-art compact and high-performance electronics, the amplification of electronic signals is an area where micro-/nanomechanics has not experienced much progress. In contrast to semiconductor devices, the performance of the proposed nanoelectromechanical amplifier improves significantly as the dimensions are reduced to the nanoscale presenting a potential pathway toward deep-nanoscale electronics. The nanoelectromechanical amplifier can also address the need for ultranarrow-band filtering along with the amplification of low-power signals in wireless communications and certain sensing applications, which is another need that is not efficiently addressable using semiconductor technology.

## Introduction

MEMS resonators have drawn the attention of many researchers over the past two decades in hopes of enabling highly integrated on-chip timing and frequency selection. The ‘resonant gate transistor’ demonstrated in 1967 (Ref. 1), which is known to be the first MEMS resonator, was an active device that integrated a relatively high mechanical Q of a suspended metallic cantilever within a field-effect transistor (FET). However, the majority of recent research on micromechanical resonant devices has focused on piezoelectric and capacitive resonators that are only passive electrical components. Such devices have not been able to meet the selectivity and pass-band loss requirements for wireless communications, leading researchers to turn their focus back to active on-chip resonant components, such as the resonant amplifier, which uses capacitive actuation and piezoresistive readout^2^. Recently, efforts were made to further combine the benefits of N/MEMS resonators with FETs. The resonant beam was selectively doped for field effect transduction, which is similar to a FET^3^; flexural beam NEMS resonators were integrated with FinFETs^4^; unreleased resonant body transistors were incorporated with *n*-channel FETs for piezoresistive sensing^5^; AlGaN piezoelectric transduction capability in combination with 2DEG electron gas at the AlGaN/GaN interface were utilized as the conducting channel for a high mobility field effect transistor^6^; and the motion sensing of a piezoelectric resonator in close proximity to a fabricated silicon nanochannel FET^7^.

This study took such efforts a major step further by taking advantage of the previously demonstrated internal thermal-piezoresistive energy pumping^8^, which was used primarily to drive self-sustained mechanical oscillation, to reach significantly higher amplification and effective quality factors. Using the proposed method, signal amplification can be delivered through a very simple device that offers extreme miniaturization potential without using field effect technology for readout. These capabilities are vital in numerous areas, such as communication systems and sensing applications. In a superheterodyne structure, for example, the device presented in this study can potentially reduce the number of stages of amplification and filtering that are primarily designed to counter the effects of a low quality factor of on-chip elements. Such a device can also be used at the output of a sensor to reduce the noise using ultranarrow-band filtering and amplifying the signal concurrently to increase the minimum detecting capability of the sensor. In addition to frequency selection, it is shown that scaling down the dimensions into the low nanoscale range can improve the performance of such devices.

## Operation principle

A piezoresistor is an active transducer by nature because it can absorb electrical power from a direct current (DC) bias source and transfer modulated power to a load. Similar to the current flow in a MOS transistor channel that is controlled by an input gate voltage, the current in a piezoresistor is controlled by its mechanical stress. Therefore, an electrical component very similar to a transistor called the ‘electromechanical transistor’ can be created by combining a piezoresistor with an electromechanical actuator, turning an input control voltage into a mechanical force.

The piezoresistive effect, however, is generally far too weak for such an electromechanical transistor to offer a useful amplification factor (that is, a gain >1). For example, although the current in a solid-state transistor can be modulated over its full range by only a fraction of a volt, 1 MPa of mechanical stress changes the current in a silicon piezoresistor by <0.1%. However, there are ways to tackle this weakness and create an electromechanical transistor that is capable of significant amplification. One convenient approach to improve the performance of the electromechanical piezoresistive transistor is to use mechanical resonance to achieve displacements that are orders of magnitude larger and consequently mechanical stress with the same amount of electrically generated input mechanical force. A resonant mechanical structure that is excited into vibration in a resonant mode with quality factor of *Q* will have a *Q*-times-larger vibration amplitude and therefore stress amplitude compared with when the same structure is subjected to the same force magnitude applied as a constant (that is, DC) force.

**Figure 1:**
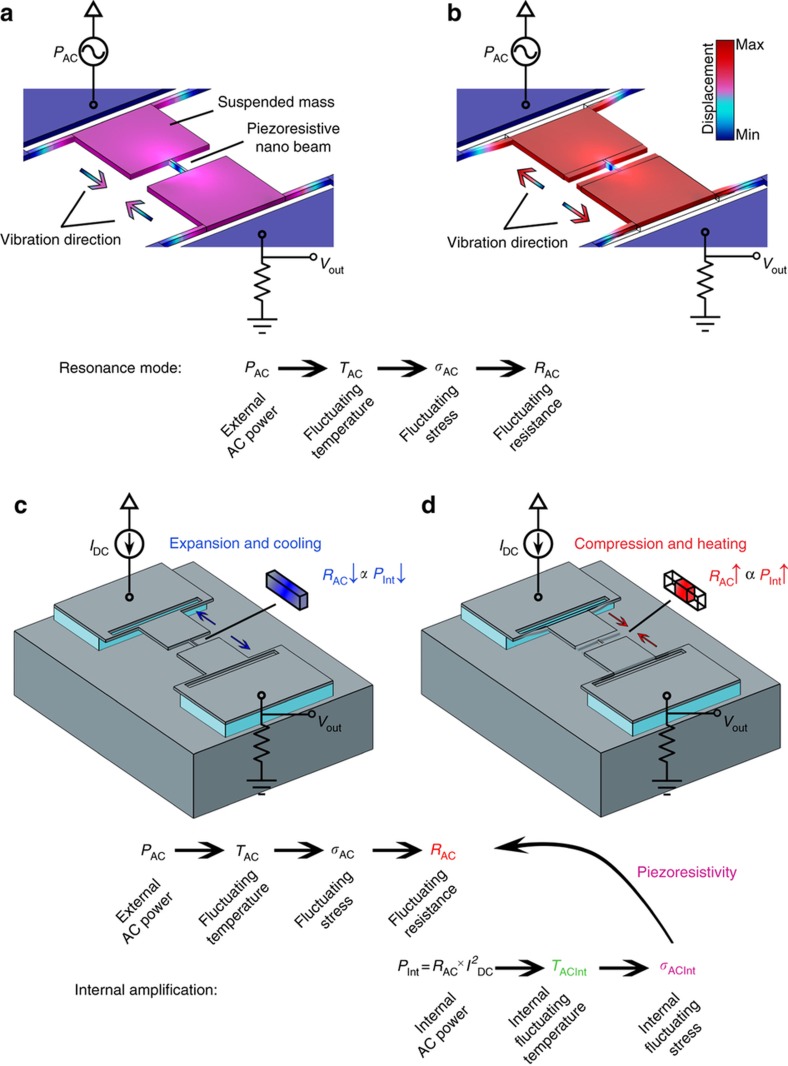
Operation principle of the electromechanical resonant amplifier with internal amplification effect. (**a**) As the fluctuating input AC power decreases, the piezoresistive beam contracts as a result of its decreasing temperature and therefore longitudinal stress. (**b**) Increasing the fluctuating input power causes the piezoresistive beam to expand due to Joule heating and therefore thermal expansion. (**c**) Additional force toward compression as the piezoresistive beam expands and cools concurrently due to the reduction in the piezoresistor value, when biased with a constant DC bias current. (**d**) Additional force towards expansion as the beam contracts due to an increase in the resistance and therefore the internal heat.

In addition, a piezoresistor can also act as a thermal actuator, generating the force for excitation of its mechanical resonance via Joule heating. Alternating current (AC) power applied to a piezoresistor can create a relatively large vibration amplitude and therefore significant stress fluctuations (*σ*_AC_) via Joule-heating-induced thermal expansion and contraction, if it has a frequency near the mechanical resonant frequency of the piezoresistor structure. This alternating stress modulates the resistance (*R*_AC_) of the piezoresistor that is now also acting as a thermal actuator. Two relatively large masses added to the two ends of the piezoresistor lower the resonance frequency of the overall structure, thus allowing the thermal response of the actuator to have a time constant closer to the mechanical resonance period. The full structure of the resonant electromechanical amplifier is shown in Figures 1a and b.

The internal amplification effect, which is the primary focus of this study, originates from the fact that the electrical resistance of the thermal actuator is itself modulated by the vibrations. It is a self-amplifying effect that results from the coupling of the thermal actuation forces and the piezoresistivity of the piezoresistor. The alternating resistance of the piezoresistor *R*_AC_ created by the external AC power at resonance along with a DC current can create a new source of internal AC power that can amplify the vibration amplitude even further, if it is in phase with the externally applied AC power. For this to occur, this additional heating power needs to decrease when the piezoresistor is expanding during resonance so that the actuator begins cooling to help contract the piezoresistor, as shown in Figure 1c. In the same manner, the piezoresistive heating power created by the DC bias current needs to increase while the actuator is contracting to help the actuator expand again during the next half-cycle, as shown in Figure 1d. The electrical resistance of a piezoresistor with a negative piezoresistive coefficient (for example, n-type doped single-crystalline silicon) decreases upon expansion and increases due to compression. Therefore, when biased with a constant current, a negative piezoresistor can provide the correct phase for the internal power (*P*=*R*_AC_×*I*_DC_^2^) to help amplify the vibration amplitude in the electromechanical resonant amplifier. In this manner, the piezoresistor absorbs energy from the DC bias source and amplifies the mechanical vibration amplitude and, along with it, the stress in the piezoresistor. As a result, the voltage fluctuations across the piezoresistor (that is, the resonant electromechanical amplifier output voltage) will be amplified.

## Modeling

The electromechanical resonant amplifier can be modeled as two parallel elements: a passive resistance that represents the physical electrical resistance of the piezoresistor, and a series RLC tank that represents the motional resonant behavior of the device. In this configuration, the current passing through the RLC tank represents the motional current of the resonant structure or the modulated bias current due to vibration-induced piezoresistive fluctuations. The motional conductance (*g*_m_) of the device is defined as the ratio of the motional current to the actuation voltage and can be approximated by Equation (1), where *α, π*_l_, *E*, and *Q*_m_ are the thermal expansion coefficient, longitudinal piezoresistive coefficient, Young’s modulus of the structural material, and the mechanical quality factor of the structure, respectively. In addition, *C*_th_ is the thermal capacitance of the piezoresistor, *ω*_0_ is the mechanical resonance frequency, and *I*_DC_ is the DC bias current^9^:

(1)gm=αEπlQmIDC2Cthω0

**Figure 2:**
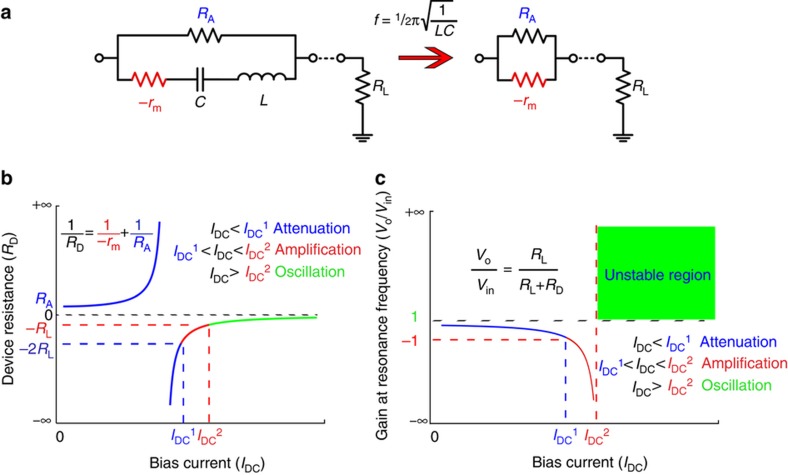
Electrical model and behavior in response to DC bias current. (**a**) Electrical model of the electromechanical resonant amplifier, consisting of the physical resistance of the piezoresistive beam (*R*_A_) in parallel with the RLC components, in which *r*_m_ is the motional resistance with negative value for a beam with a negative piezoresistive coefficient, *L=Qr*_m_*/ω*_0_, and *C*=1/(*Qr*_m_*ω*_0_). At the resonance frequency *ω*_0_, the circuit reduces to the combination of the two resistors: *R*_A_, which is the passive component, and *r*_m_, which is the active component. (**b**) Effect of the DC bias current on the overall resistance of the device at resonance frequency. By increasing the bias current and thus |*g*_m_|, the device transitions from being a passive element (that is, positive resistance values) to an active element (that is, negative resistance values). (**c**) Effect of bias current on the voltage gain. At small currents, the device is passive and thus attenuates the input signal. At marginally higher currents, the device continues to attenuate because the internal amplification is not sufficiently strong to compensate for most of the loss in the system, even though the device is in the active region (blue). At currents larger than *I*_DC_^1^, the device will amplify the input signal because the loss due to physical resistance is compensated for via internal amplification (red). At currents higher than *I*_DC_^2^ when the internal amplification has compensated for all losses in the resonator and the load, the combination will enter the oscillation zone and will become unstable (green).

The motional resistance *r*_m_, which is equal to 1*/g*_m_, can be negative or positive depending on the sign of the piezoresistive coefficient. Figure 2a shows the electrical equivalent circuit model of the resonant transistor that can form an electronic amplifier when connected to a load resistance at the output node. At the resonance frequency, the imaginary impedance of the series *L* and *C* will cancel each other out, and the model reduces to two parallel resistors: *R*_A_, which is the passive physical resistance of the piezoresistor, and *r*_m_, which is the negative motional resistance or the active element. At very small DC bias currents, *g*_m_ has a small but negative value and increases in magnitude as the current increases. Thus, as the bias current increases, *r*_m_, which initially was a large negative resistor, becomes smaller. Therefore, the overall resistance of the device *R*_D_, which is defined as the parallel combination of both physical and motional resistances (that is, *R*_D_=*R*_A_ || *r*_m_), depends on the DC bias current. On the basis of the value of *r*_m_ with respect to *R*_A_, *R*_D_ can be negative or positive, as shown in Figure 2b. If the motional resistance is greater than the physical resistance, then the overall device resistance has a positive value that is larger than or equal to *R*_A_.

Therefore, the device behaves as a passive element (that is, positive resistance). In this region, the voltage gain is defined as the voltage transferred from the input to the load resistance *R*_L_ through the device and will be positive and smaller than unity; thus, Gain=*R*_L_/(*R*_L_*+R*_D_). As the motional resistance supersedes the physical resistance in magnitude, the overall device resistance becomes negative, and the device becomes active. At this point, the internal amplification effect adds sufficient power to compensate for the power that is lost throughout the physical resistance of the device. However, because *R*_D_ remains large, the voltage gain is less than unity, and the input voltage remains attenuated. Up to this point, the device has been in attenuation mode with regard to the bias current, as shown by the blue color in Figure 2. As the bias current increases, the device resistance decreases until it reaches the value of twice the load resistance (−2*R*_L_), where the voltage gain becomes unity (Gain=−1). Then, the internal amplification has compensated for all power losses and converts the power it takes from the DC source into a modulated output voltage that is equal to the input; this is the onset of the amplification mode, which is identified by the red color in Figure 2. The negative sign in Figure 2c represents a 180° phase shift of the signal from the input to the output. As the bias current increases further, the device continues to amplify the signal with larger gains until the device resistance also becomes smaller than the load resistance (*R*_D_*=*−*R*_L_); then, the internal amplification compensates for all losses throughout the system, including the load. Thus, the overall combination becomes unstable, initiating a self-sustained oscillation^10^. The gain then becomes positive again with a value larger than unity, indicating an amplifying positive-feedback loop (that is, an oscillator). Therefore, the vibration amplitude continues to increase until it is limited by the mechanical saturation of the piezoresistive beam. The oscillation mode is shown in green in Figure 2.

## Fabrication of electromechanical amplifiers and measurements

**Figure 3:**
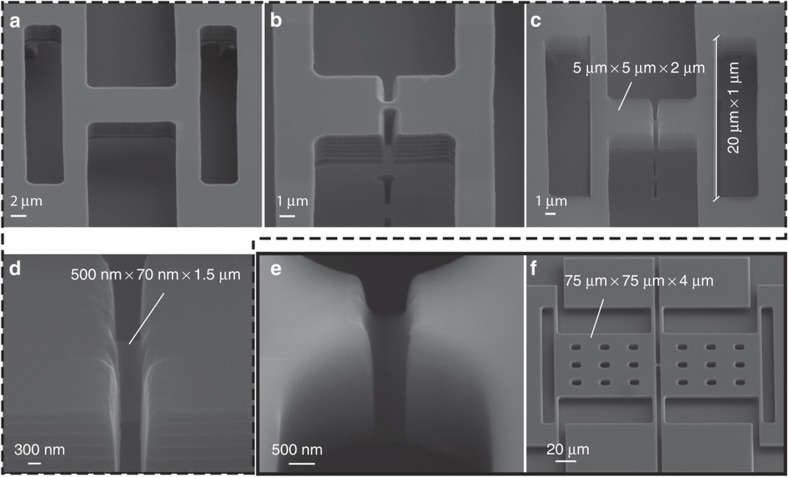
SEM images of the nanoelectromechanical amplifier after each fabrication step. (**a**) Initial shape of the device fabricated on an SOI wafer with n-type doped, 0.04-Ωcm resistivity and 2-μm device layer after patterning of the support beams and a single mass using photolithography followed by deep reactive ion etching (DRIE) and HF removal of the buried oxide layer. (**b**) The center of the initial single mass is etched from the sides using FIB to form the two suspended masses along with a preliminary wide beam in the middle. (**c**) The final image of the fabricated device after FIB etching continues with a higher precision to form a 100-nm-wide piezoresistive beam and successive dry thermal oxidation and oxide removal in HF to trim the edges and reduce the beam width to 70 nm. (**d**) Close-up image of the final beam. (**e** and **f**) Close-up image of the beam and the final fabricated device, respectively, of a similar beam with larger masses (75 μm×75 μm), which provide gain at lower frequencies.

Fabrication of the proposed nanoelectromechanical amplifiers begins with lithography on a silicon-on-insulator (SOI) wafer composed of a thin silicon device layer that is typically 2-μm thick and is separated from a silicon handle layer by a silicon dioxide thin film that is 2-μm thick. The support beams and a rectangular pattern the size of the overall device are then etched into the device layer using deep reactive ion etching (DIRE). The structure is suspended by removing the underlying insulating SiO_2_ layer in hydrofluoric acid (Figure 3a). At this stage, a preliminary beam is created by etching the initial rectangular shape of the device to create the masses, which are 5 μm×5 μm, using a focused ion beam (Figure 3b). The preliminary beam is then thinned down further to ~100 nm, and eventually, the desired beam width of 70 nm is achieved by consecutive dry thermal oxidation and oxide removal in hydrofluoric acid, as shown in Figures 3c and d. A second device with similar beam dimensions that operates at a lower frequency with larger masses (that is, 75 μm×75 μm) was also fabricated. The SEM image of the second device is shown in Figures 3e and f.

## Results

**Figure 4:**
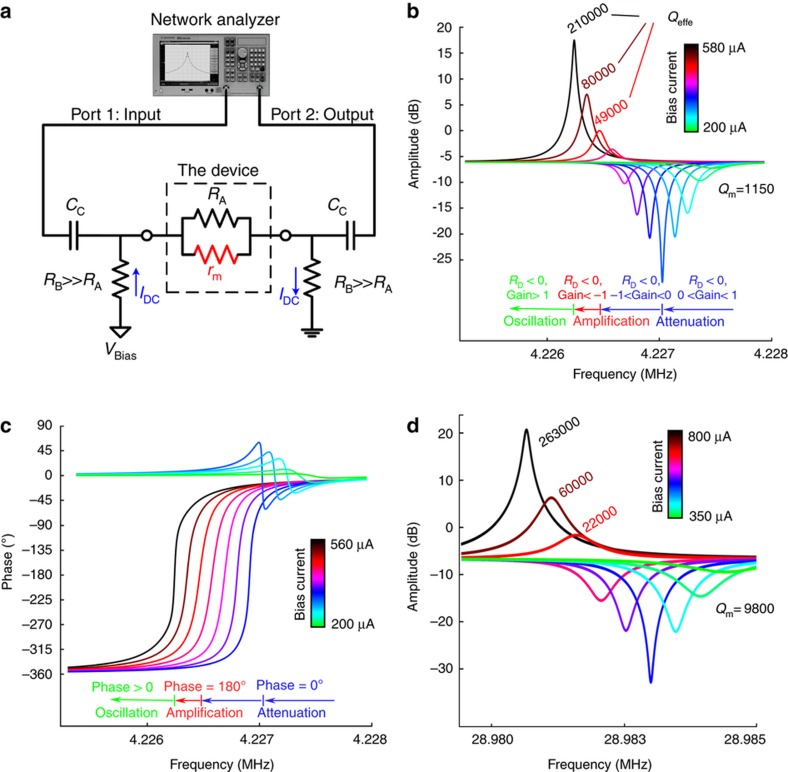
Measured frequency response of the nanoelectromechanical resonant amplifier. (**a**) Measurement setup, showing the connection between the network analyzer and the device. (**b**) amplitude and (**c**) phase response of a nanoelectromechanical resonant amplifier, operating at 4.2 MHz measured by a network analyzer for a matched load of *R*_L_*=R*_A_. By increasing the DC bias current, the device makes the transition from attenuation to amplification and finally to oscillation at the resonance frequency, as predicted in Figure 2. At low bias currents, where the device is passive, the phase is zero with an absolute voltage gain of less than one. At the transition from the passive region to the active region, where the device resistance *R*_D_ is infinite, it is shown that the gain has the strongest downward peak, indicating that little of the input signal passes through the device to the load. In the active region, the peak begins to grow towards amplification with a phase of 180°. On the verge of oscillation, the phase becomes zero again, and the system becomes unstable. (**d**) Measured frequency response of a 30-MHz electromechanical amplifier, which shows a similar trend.

As shown in Figure 4a, a network analyzer was used to measure the frequency response of the devices. A constant DC current controlled by a voltage source and large bias resistors relative to the device was established through the piezoresistor. The input/output ports of the network analyzer were then connected through the coupling capacitors. Figures 4b and c show the measured frequency response of the electromechanical amplifier with mass plate dimensions of 4 μm×75 μm×75 μm and beam dimensions of 70 nm×500 nm×2 μm with a resonance frequency of 5 MHz. At low bias currents, where the device is operating in attenuation mode and the device resistance is positive, the downward peak becomes stronger (that is, more attenuation) because *R*_D_ increases at resonance frequency with bias current, while the input-to-output phase change is zero. As the device becomes active but is still in attenuation mode, the downward peak begins to move up in the gain amplitude because the internal amplification compensates for the loss through the physical resistance and delivers more power to the load, while the phase remains 180°.

As the voltage gain increases, the effective quality factor (*Q*_eff_), which is also defined as the ratio of the resonant frequency to the −3 db bandwidth (*f*_o_ */∆f*_–3db_), increases. The effective quality factor differs from the mechanical quality factor *Q*_m_ used in Equation (1). Although the mechanical quality factor, which is defined as the ratio of the mechanically stored energy to the energy lost per cycle, which is also determined by the structural properties of the device, remains constant, an increase in the bias current will result in the compensation for the loss as a product of internal amplification (that is, addition of stored energy created by the electromechanical coupling) and therefore a higher effective quality factor. The increase in the gain at resonance frequency (that is, a higher peak value) manifests itself in a smaller bandwidth, thereby resulting in a sharper upward peak and a higher effective quality factor.

The measured frequency response of the higher-frequency (for example, 30 MHz) electromechanical amplifier with smaller masses of 5 μm×5 μm×2 μm (Figure 4d) exhibits similar behavior. As predicted by Equation (1), a higher bias current is required to obtain the same gain from a higher frequency device with the same piezoresistor dimensions because a lower mass yields a lower *g*_m_ when other parameters are held constant.

Table 1 shows the parameters obtained from the measurement. In theory, the resonance frequency must remain constant with changes in the bias current. However, as the bias current increases due to increases in DC temperature of the piezoresistor and the temperature dependence of Young’s modulus, the stiffness of the beam decreases and leads to a marginal shift of the resonant peak towards a lower frequency.

## Discussion

### Scaling and performance improvement

Lowering the bias current required to maintain the gain of an electromechanical amplifier is critical to its low-power operation. Aside from the overall significance of a desired low-power consumption for any electronic device, a high DC power consumption typically leads to excessive heating and reliability issues, a reduction of the piezoresistive coefficient^11–13^, and higher levels of thermal and mechanical noise. Therefore, optimization is required for such devices to maintain high levels of gain while lowering the required DC power.

One method to optimize performance and lower the DC power required is to reduce the electrical resistivity of the piezoresistive beam. If the resistivity is lowered by a factor of *α*, then the bias current can be increased until the *g*_m_ is *α* times larger without changing the DC power consumed in the beam (*P*_DC_ ∝ *α*^−1^*R*_A_*. αI*_DC_^*2*^ and *g*_m_ ∝ *αI*_DC_^*2*^). Therefore, for the same amount of power, an *α*-times-lower device resistance *R*_D_ and consequently a larger gain can be achieved by lowering the resistivity.

However, the most efficient way to optimize the performance of the nanoelectromechanical amplifier is to scale down its physical dimensions. Assuming that a given piezoresistive coefficient *π*_l_, the mechanical quality factor *Q*_m_, and Young’s modulus *E* remain constant, the effect of scaling down the device dimensions on *g*_m_ can be predicted using Equation (1). By obtaining the parameters from measurements in Figure 4 at constant bias currents and the physical dimensions of the device shown in Figure 3, the transconductance value of the scaled device *g*_ms_ can be found from Equation (2), where *S*_L_, *S*_A_, and *S*_M_, which have positive values less than unity, are scaling factors for the piezoresistive beam length, the beam cross sectional area and the suspended masses, respectively. To scale down the device and maintain a constant resonance frequency, the suspended masses must be scaled down by the same factor as the longitudinal stiffness of the piezoresistive beam (that is, *S*_M_*=S*_A_*/S*_L_). With this method, Equation (2) reduces to $g_{\mathrm{ms}}\propto \frac{1}{S_{A}S_{L}}$, suggesting that the performance can be improved for a constant frequency.

If the beam length, then beam area and masses are scaled down together (that is, *S*_L_*=S*_A_*=S*_M_*=S*), the transconduction *g*_ms_ will improve by a rate of *S*^−3/2^, whereas the resonance frequency will increase by a rate of *S*^−1/2^, indicating that higher transconduction values can be achieved at higher frequencies:

(2)gms=gmCthω0Cthsω0s=gmSMSASASL

This method of scaling will also improve the DC temperature and power consumption in the piezoresistive beam because the thermal and physical resistance of the beam remain constant when scaling the beam length and cross sectional area concurrently. Thus, it can be argued that by scaling the device down, higher levels of gain can be attained without increasing the power or DC temperature in the piezoresistive beam.

As stated earlier, the effective quality factor, which is defined as the ratio of the resonant frequency to the −3 db bandwidth (*f*_o_*/∆f*_-3db_), increases with internal amplification. Equation (3) shows an approximation for the effective quality factor that is only valid when the device is in the amplifying region. For the proof of Equation (3), please refer to the Supplementary Information. In this equation, *Q*_m_ is the mechanical quality factor; *r*_m_ is the motional resistance or 1/*g*_m_; and *R*_A_ and *R*_L_ are the physical and load resistances, respectively. As the negative parameter *r*_m_ decreases from −2*R*_L_ to *–R*_L_||*R*_A_, the denominator approaches zero, and the effective quality factor increases markedly:

(3)Qeff=Qm(1+RL‖RArm)2+(1+RL‖RArm)2

As discussed, scaling the dimensions of the device will reduce the absolute value of the motional resistance for a constant bias current by a rate of *S*^1.5^. Therefore, scaling the dimensions of the device will produce the same effective quality factors for smaller bias currents because the term $\frac{R_{L}\|R_{A}}{r_{m}}$ (typically$\approx \frac{R_{L}}{r_{m}}$) in Equation (3) approaches negative unity at a lower power.

When the resonant amplifier is near oscillation (that is, *g*_m_ is near the *g*_L_*+g*_A_) any level of gain and effective quality factor can be achieved in theory without significantly changing the DC power. However, as the gain increases, the bandwidth decreases. Near oscillation, the bandwidth is sufficiently small that the device can be easily pushed into its unstable region by a small perturbation, limiting the maximum level of gain achievable in practice.

**Figure 5:**
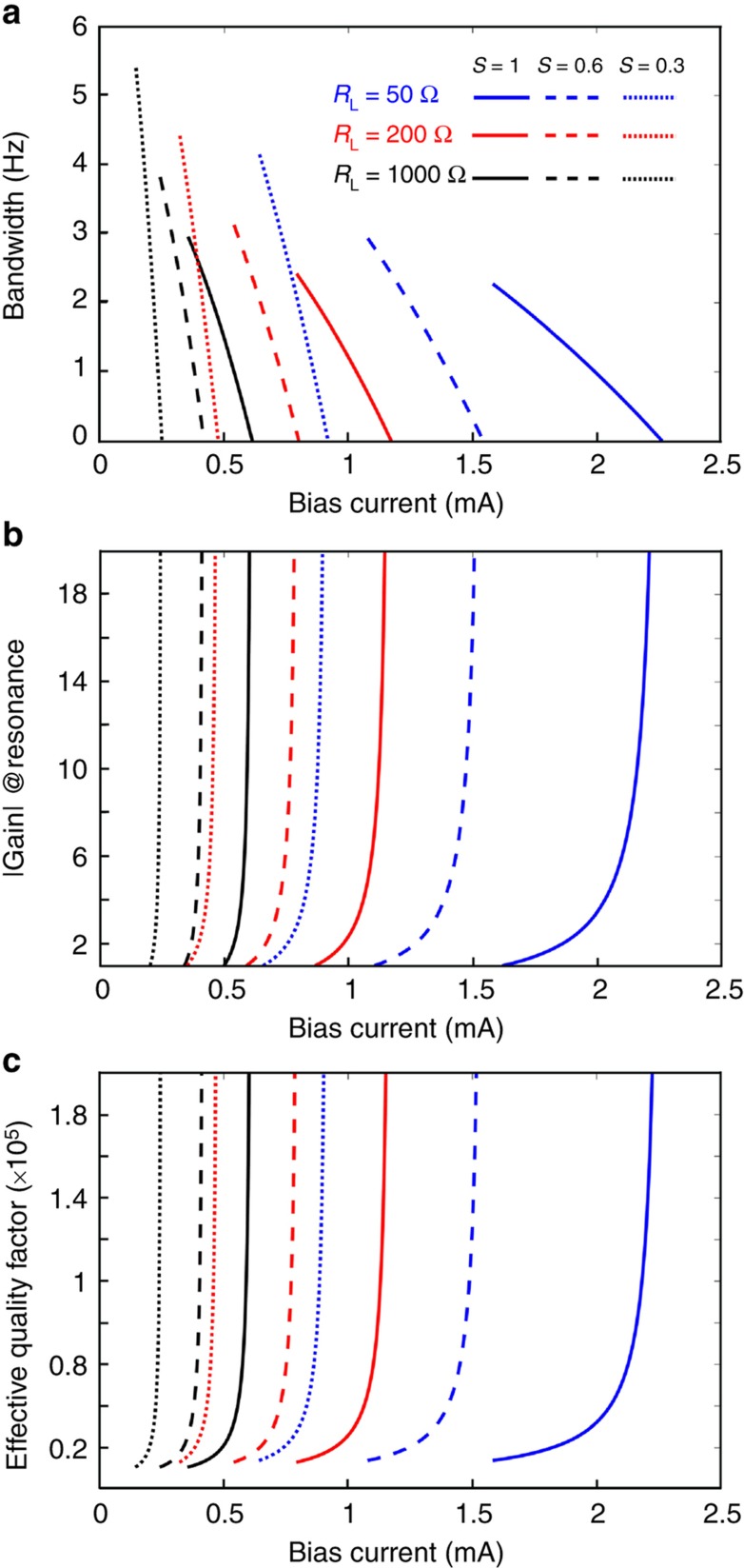
Effect of scaling down the nanoelectromechanical amplifier. (**a**) Trend of change in bandwidth by increase in bias current for different loads (solid lines), and the effect of scaling on the bandwidth (dashed line for *S*=0.6, and dotted line for *S*=0.3) due to the increase in effective quality factor. (**b**) Gain vs. bias current (solid lines) and the effect of scaling on gain (dashed line for *S*=0.6, and dotted line for *S*=0.3) (**c**) Effective quality factor achieved at lower power as a results of scaling.

On the basis of the values obtained from the measurements and Equation (3), the bandwidth, absolute value of the gain at resonance frequency, and the effective quality factor are plotted as functions of DC bias current in the amplification region for 50-, 200-, and 1000-Ω loads, shown as solid lines, in Figure 5.

Figure 5 shows that by scaling all dimensions (that is, beam length, area, and masses) concurrently with the rates of *S*=0.6 and 0.3, which are represented by the dashed and dotted lines, respectively, the same levels of gain and effective quality factor at lower bias currents can be achieved, demonstrating the potential for such devices that are fabricated at the low nanoscale have relatively larger gains and bandwidths at smaller powers (constant *R*_A_ and smaller *I*_DC_) and at a higher frequencies.

Although it is clear that reducing the size of the piezoresistive beam in the described resonant nanoelectromechanical amplifiers can significantly improve the device performance without fundamental limitations, the reduction of the thermal conductance and Young’s modulus of the beam are expected with device dimensions in the low nanoscale, as has been observed in nanowires^14–16^. Such effects can diminish the performance of the nanoelectromechanical amplifier by increasing the operating temperature in the beam and power dissipation and therefore reducing the mechanical quality factor. However, the large piezoresistive coefficient observed in silicon nanowires^17–19^, along with the increase in the thermal expansion coefficient^20,21^, is expected to compensate for and even improve the overall device performance.

In conclusion, even though silicon was used as the piezoresistive material in this study, the design of the proposed nanoelectromechanical narrow-band amplifier is not limited to silicon. The proliferation over the past few years of novel materials such as wide-bandgap semiconductors, including GaN and SiC, and their growing applications in RF/power electronics as well as M/NEMS^22–29^, suggests that they can create exciting opportunities and potentially replace silicon in many areas, including in the design of the device proposed in this study. They can also offer electrical and mechanical properties that match and even exceed those of silicon. Greater thermal and mechanical stability at temperatures as high as 600 °C, piezoresistive coefficients comparable to that of silicon, and higher Young’s moduli^30–33^ make these materials suitable alternatives for silicon as the basis of the nanoelectromechanical amplifier, particularly in harsh environments where silicon fails to retain its properties.

## Figures and Tables

**Table 1:** Table of parameters extracted from the measurement for each device corresponding to Figure 4

4-MHz device									
*R*_A_ (G)	1400								
*I*_DC_ (μA)	200	300	400	440	470	500	540	560	580
*L* (H)	2.330515	1.035808	0.58267	0.481568	0.422073	0.372952	0.3197614	0.2973361	0.2771902
*C* (F)	5.97E−16	1.34E−15	2.39E−15	2.89E−15	3.30E−15	3.73E−15	4.35E−15	4.68E−15	5.02E−15
*f*_0_ (Hz)	4 270 390	4 270 280	4 270 060	4 269 840	4 269 620	4 269 510	4 269 290	4 269 180	4 269 070
*r*_m_(Ω)	−6250.000	−2777.778	−1562.500	−1291.322	−1131.734	−1000.000	−857.33882	−797.19388	−743.1629
									
29-MHz device									
*R*_A_ (G)	2000								
*I*_DC_ (μA)	380	500	550	570	600	620	700	750	800
*L* (H)	0.253508	0.146429	0.121018	0.112676	0.101692	0.095239	0.0747149	0.0650861	0.0572056
*C* (F)	1.19E−16	2.06E−16	2.49E−16	2.68E−16	2.97E−16	3.17E−16	4.04E−16	4.63E−16	5.27E−16
*f*_0_ (Hz)	28 999 500	28 999 000	28 998 500	28 998 000	28 997 500	28 997 000	28 996 500	28 996 000	28 995 500
*r*_m_ (Ω)	−4616.805	−2666.667	−2203.857	−2051.913	−1851.852	−1734.305	−1360.5442	−1185.1852	−1041.6667
