# Supplementary material for: Nanoelectromechanical resonant narrow-band amplifiers
Source: Microsyst Nanoeng. 2016 Mar 24;2:16004. doi: 10.1038/micronano.2016.4 (PMC6444725; doi:10.1038/micronano.2016.4)
Supplement: Supplementary Information [file micronano20164-s1.pdf]

Supplementary file

Nanoelectromechanical resonant narrow-band amplifiers

METHODS

Measurement

All measurements in this study were performed under a 1-mtorr vacuum. The frequency responses of the nanoelectromechanical device were measured using a network analyzer with a load of 50 Ω, and the results were mapped to a matched load of $R_L = R_A$, as reported in Figure 4.

Derivation of bandwidth

On the basis of the model described in Figure 2a, the transfer function of the nanoelectromechanical amplifier can be written as:

$$S_{21} = \frac{R_L}{Z_d + R_L} = \frac{R_L}{\frac{LCR_A s^2 + R_A r_m Cs + R_A}{LCs^2 + (r_m + R_A)Cs + 1} + R_L} \tag{S1}$$

where Z_d is the impedance of the device including both physical and electromechanical components, and r_m is the motional resistance. By replacing L and C with the values Qr_m/ω_0 and $1/(Qr_m\omega_0)$, respectively, this equation can be simplified to:

$$S_{21} = \frac{R_L}{(R_A + R_L)} \frac{s^2 + \frac{\omega_0}{Q_z}s + \omega_0{}^2}{s^2 + \frac{\omega_0}{Q_p}s + \omega_0{}^2} \tag{S2}$$

where $Q_z = Q_m \frac{r_m}{r_m + R_A}$, and $Q_p = Q_m \frac{r_m(R_A + R_L)}{R_A r_m + R_L(r_m + R_A)}$. For the amplification region where the peak is upward, and the gain $|S_{21}\ (j\omega_0)|$ is larger than unity for frequencies located within the bandwidth, Equation (S2) can be approximated as:

$$S_{21} = \frac{R_L}{(R_A + R_L)} \frac{\frac{\omega_0}{Q_z}s}{s^2 + \frac{\omega_0}{Q_p}s + \omega_0{}^2}$$

Eventually, the bandwidth can be calculated by solving for $S_{21}\ (j\omega_0 + \Delta\omega) = S_{21}\ (j\omega_0)/\sqrt{2}$ and can be approximated as:

$$2\Delta\omega \approx \frac{\omega_0}{Q_p}\sqrt{2 + \frac{1}{Q_p{}^2}} \tag{S3}$$
